# Bud-Localization of *CLB2* mRNA Can Constitute a Growth Rate Dependent Daughter Sizer

**DOI:** 10.1371/journal.pcbi.1004223

**Published:** 2015-04-24

**Authors:** Thomas W. Spiesser, Clemens Kühn, Marcus Krantz, Edda Klipp

**Affiliations:** Theoretical Biophysics, Humboldt-Universität zu Berlin, Berlin, Germany; Fondazione Edmund Mach, Research and Innovation Centre, ITALY

## Abstract

Maintenance of cellular size is a fundamental systems level process that requires balancing of cell growth with proliferation. This is achieved via the cell division cycle, which is driven by the sequential accumulation and destruction of cyclins. The regulatory network around these cyclins, particularly in G_1_, has been interpreted as a size control network in budding yeast, and cell size as being decisive for the START transition. However, it is not clear why disruptions in the G_1_ network may lead to altered size rather than loss of size control, or why the S-G_2_-M duration also depends on nutrients. With a mathematical population model comprised of individually growing cells, we show that cyclin translation would suffice to explain the observed growth rate dependence of cell volume at START. Moreover, we assess the impact of the observed bud-localisation of the G_2_ cyclin *CLB2* mRNA, and find that localised cyclin translation could provide an efficient mechanism for measuring the biosynthetic capacity in specific compartments: The mother in G_1_, and the growing bud in G_2_. Hence, iteration of the same principle can ensure that the mother cell is strong enough to grow a bud, and that the bud is strong enough for independent life. Cell sizes emerge in the model, which predicts that a single CDK-cyclin pair per growth phase suffices for size control in budding yeast, despite the necessity of the cell cycle network around the cyclins to integrate other cues. Size control seems to be exerted twice, where the G_2_/M control affects bud size through bud-localized translation of *CLB2* mRNA, explaining the dependence of the S-G_2_-M duration on nutrients. Taken together, our findings suggest that cell size is an emergent rather than a regulatory property of the network linking growth and proliferation.

## Author Summary

The size between different organisms ranges considerably, yet, the size of the individuals and even the same types of cells within the individuals are remarkably constant. Cell size emerges from the balance between how fast the cell grows and the frequency with which it divides. This system level coordination of growth and division is universal across species and is required to ensure faithful duplication and genetically intact offspring. We have devised a computational model for the interplay of growth and division in the premier model organism, Baker’s yeast, to test the fundamental architecture of this coupling and to assess the role that cell size itself can play in it. In contrast to traditional theories that assume a yet-to-be-determined cell size sensor, our model relies on a single mechanism, effectively measuring the cell’s translational capacity, applied twice at different stages of the cell’s life-cycle to explain this coupling. In our model, a growth condition specific cell size emerges, as has been found in experiments. Our analysis shows how the nature of the two linked properties growth and proliferation can shape eukaryotic cells and explain cell size as an emergent rather than regulatory property of this process.

## Introduction

Cell size is a fundamental systems level property of life. It emerges as a combination of the cell cycle, controlling the orderly orchestration of duplication and division, and the individual growth rate, reflecting extra- and intracellular physiological conditions. The cell cycle and the growth rate are coupled, such that proliferation and growth are balanced, avoiding abnormally large or small cells. Understanding the coupling is of particular interest for two reasons. First, the cell cycle as well as cellular growth are two fundamental properties that can be found in nearly all forms of life. Second, decoupling of the two can have disastrous consequences for an organism, e.g. deterioration of cell size.

The unicellular eukaryote *Saccharomyces cerevisiae* can be observed to grow to a ‘critical cell size’ in the G_1_ phase before committing to passage through the cell cycle [1]. The commitment is called START in *S. cerevisiae* and constitutes the transcriptional activation of more than 200 genes by the transcription factor complexes SBF and MBF [2]. This triggers the onset of downstream events, such as budding and DNA replication. SBF/MBF activity is controlled by the G_1_ network, which involves the cyclin dependent kinase (CDK) Cdc28, its activating subunits the G_1_ cyclins Cln1/2/3 and the transcriptional repressor Whi5 (reviewed in [3]). The most upstream undisputed activator of START is Cln3. Cln3 binds to and activates the CDK to phosphorylate Whi5, which relieves the repression of SBF/MBF. The START transition is triggered when a critical activity of the CDK is reached [4]. Beyond the critical level, CDK activity stabilises through positive feedback involving Cln1/2 [5, 6]. The core network architecture with the competition between the active CDK and the transcriptional repressor is analogous to the Restriction Point, which is the equivalent of START in mammalian cells [7]. The nature of the mechanism within the START network that ties growth and proliferation together remains unknown.

Size control must be as old as the cell cycle itself. It is conserved across species over a huge range of cell sizes and shapes, and it is well established that size control can occur in cell cycle phases other than G_1_ [8]. Recent evidence strongly suggests that also in budding yeast size control is likely to be exerted outside of G_1_ [9, 10]. The fission yeast *Schizosaccharomyces pombe* has a size control checkpoint at the G_2_/M boundary and many of its components are conserved in budding yeast [11, 12]. The observation that the budded phase duration responds to growth media and the high degree of conservation between the two yeasts prompts the question, whether a size control mechanism guards mitotic entry in budding yeast as well [13–16]. Unfortunately, size control at the G_2_/M transition is less well understood in budding yeast [8, 9, 17–19].

It is well known, however, that *S. cerevisiae* can arrest its cell cycle at the G_2_/M boundary through activation of the so called morphogenesis checkpoint [20]. The kinase responsible for mitotic entry Clb2-Cdc28 is inhibited through phosphorylation at the Tyr19 residue by Swe1 [20, 21]. Re-activation of Clb2-Cdc28 requires removal of the inhibitory phosphate by the mitotic inducer homolog Mih1 phosphatase [22]. The exact property that is monitored in budding yeast remains a matter of debate, but it has been argued that the checkpoint responds to perturbations of the actin cytoskeleton or even bud growth [9, 17, 18]. Indeed, recent work suggests that polarised exocytosis may be required to pass through the checkpoint [9]. This hypothesis directly connects membrane growth at the bud site to cell cycle progression suggesting a growth dependent size control checkpoint for mitotic entry in budding yeast [9].

In the regulation of both the START and the G_2_/M transitions, a master CDK is balanced against an opposing regulator that must be overcome to initiate crucial cellular events, like DNA replication or cell division [3]. The master CDK is activated in G_1_ and G_2_ by the accumulation of cyclins and this activation is antagonised by CDK inhibitors and rapid degradation of the cyclins to form elaborate molecular switches [23, 24]. Accordingly, the transitions occur in a switch like manner, when the time (or size) is right. It is not exactly clear how growth can flip a transition switch, but one theory for size control proposes the level of an unstable cell cycle regulator as a gating device to measure the growth capacity of the cell [25, 26]. The growth or biosynthetic capacity of the cell determines the growth rate and the unstable regulator is presumed to be one of the G_1_ cyclins, most likely Cln3 [8]. Cln3 levels are heavily influenced by the available nutrients and Cln3 translation is slowed down in conditions when fewer ribosomes are available [27–29]. The G_1_ cyclins and other components of the START network modulate cell size at START [15, 30]. Additionally, many other components influence cell size, especially those with a functional link to the cellular growth machinery, like Sfp1 or Sch9 [31]. Recently, it was shown that cell size at START is set as a function of the individual growth rate of a cell and that START network components modulate the strength of this correlation, arguing for the existence of a growth rate dependent cell sizer in G_1_ [15].

Both *in vivo* and *in silico* analysis suggested that G_1_ is not the only size control phase during the cell division cycle [9, 10, 17, 32]. Also, if G_1_ were the sole size control phase, the question would remain how budding yeast can maintain size homeostasis and control in case the mechanisms in G_1_ are impaired, as e.g. through *CLN3* overexpression. *CLN3* overexpression mutants display a substantial reduction in G_1_ length and a small cell size (*whi* phenotype) [33, 34]. Since Cln3 is an upstream activator of START, its overexpression can intuitively explain the shortened G_1_ phase and the concomitant reduction in cell size. Counterintuitively, the generation time remains largely unchanged, arguing for the existence of another control point further downstream in the cell cycle to compensate for the reduced time in G_1_ [33, 34].

The obvious candidate for this would be the G_2_/M transition. Here, the mitotic cyclins Clb1/2 are responsible for CDK activity to trigger mitotic entry [4]. Intriguingly, *CLB2* mRNA (*mCLB2*) accumulates in the bud, while the Clb2 protein is distributed throughout the cell [35, 36]. If the cell localises its *CLB2* transcripts to the bud it is likely that translation of the transcript is also a local phenomenon. In principle, active transport of the mRNA leading to localised translation of *mCLB2* could serve to measure the biosynthetic capacity of the bud, to form a bud (daughter) sizer in G_2_. Once the bud is strong enough for independent life, measured through production of sufficient Clb2, the cell enters mitosis. It is tempting to speculate that if Cln3 is a cell sizer, then Clb2 might be a bud sizer.

Here, we approach the elusive problem of size control from a theoretical angle, and use a number of mathematical models for rigorous testing of different control concepts. Our approach is to model single cells that are capable of growth and division, and grow them in *in silico* cell cultures. Through inheritance, we let the culture evolve over time, generating fully traceable cell populations for analysis [32]. In the model, growth and proliferation is integrated through production of a critical unstable cell cycle regulator as function of the biosynthetic capacity of the cell. Using this approach, we formally establish that the accumulation of cyclins to a threshold provides the necessary prerequisites for size control and homeostasis. Since it is unlikely that size control occurs in G_1_ alone, we use models that include additional size control at the G_2_/M boundary. We find that we need to take into account the intriguing, and to our knowledge unexplained, fact that *mCLB2* is localised to the growing bud of yeast cells to fully explain experimental data [35]. Bud-localised translation of the major cyclin that activates the master CDK for mitotic entry can, in theory, constitute a growth rate dependent sizer for the bud. With the suggested mechanism we (i) offer a functional explanation for the *mCLB2* transport into the bud, (ii) elucidate the prolongation of the budded phase in response to poor nutrients or *CLN3* overexpression, and (iii) propose a unifying model for integration of growth and division in the G_1_ and G_2_ phases of budding yeast.

## Results

### Linking growth and division through cyclin translation leads to size homeostasis and a growth rate dependent sizer in G_1_

**Fig 1:**
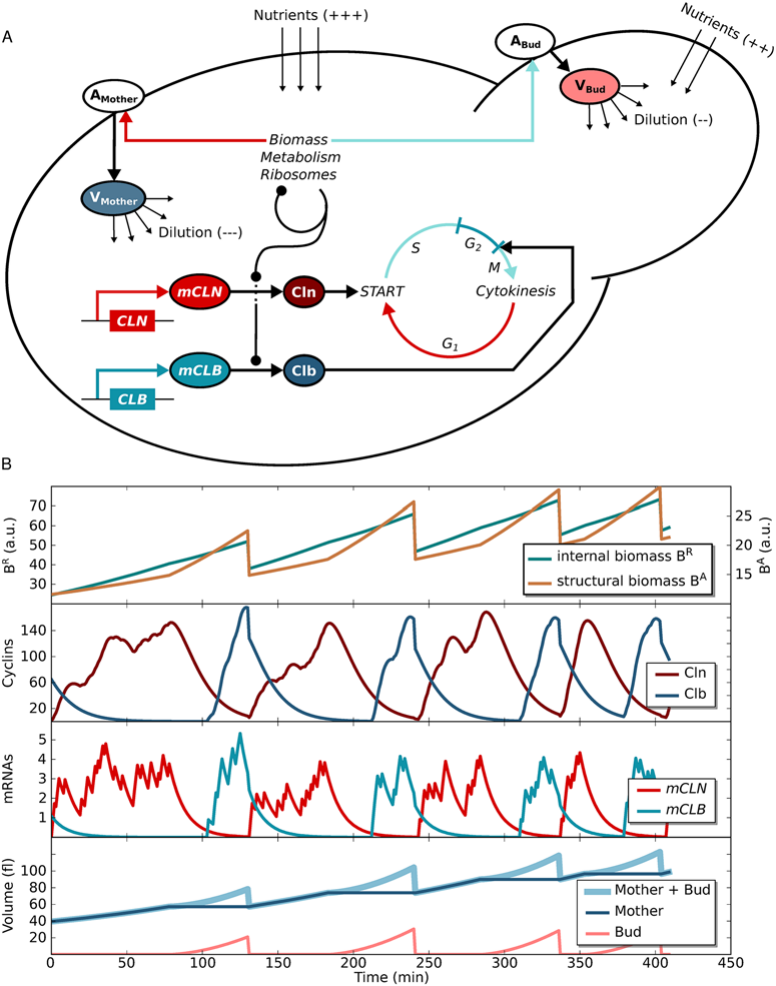
Schematic growth model and its dynamics. (A) The model produces internal biomass (ribosomes, metabolic enzymes, polymerases etc.) in a self-replicating fashion. It also accumulates structural biomass that forms the cell area (membrane and wall). Depending on cell cycle stage the area of the mother (G_1_) or the bud (S-G_2_-M) grows. Biomass dependent translation of cyclins triggers cell cycle transitions (Cln: G_1_/S; Clb: G_2_/M; S and M phases are constant). There are two model versions to distinguish translation of *mCLB* in the whole cell (Model-1) or only in the bud (Model-2). (B) Trajectories of Model-1 variables for one realization of four consecutive cell cycles starting with a new-born cell, then following the mother line. Cyclins and mRNAs are given in molecule numbers.

We present here an extended version of a minimal eukaryotic cell model that is capable of growth and division (Fig 1A) [32]. In the model, two types of biomass define growth: structural and internal biomass. Structural biomass represents all those components that are destined for the cell wall or membrane. Internal biomass are soluble agents within the cell, describing the cell’s capacity to metabolise nutrients and build new macromolecules (biosynthetic capacity). Structural and internal biomass accumulate with replicative age, leading to an increase in size over generations as observed experimentally (Fig 1B) [37, 38]. Budding yeast grows and divides asymmetrically and, therefore, growth of the mother and the bud is considered separately [39]. The volume trajectory for a single cell is biphasic with altered growth rates dependent on cell cycle stage as observed experimentally as well (Fig 1B) [15, 40–42]. A rudimentary version of the cell cycle machinery is included, with one proxy for the G_1_ cyclins (Cln) and one proxy for the mitotic cyclins (Clb) that determine CDK activity (Fig 1B) [43]. Transcription of cyclins is considered stochastic and restricted to a distinct cell cycle phase (*CLN* in G_1_, *CLB* in G_2_—Fig 1B). Growth and division are coupled via production of Cln in G_1_ and Clb in G_2_, as a function of the biosynthetic capacity of the cell. This means that the translation of cyclin mRNAs is dependent on the internal biomass (see also Materials and Methods). At division, two cells emerge from one and all soluble components are split according to the volume ratio of the mother and the bud. Through cell growth and division, we evolve an entire asynchronously growing population from one progenitor cell, as previously described [32]. The model is a comprehensible, minimal approximation of the complex process controlling duplication and separation in living eukaroytes [44].

**Fig 2:**
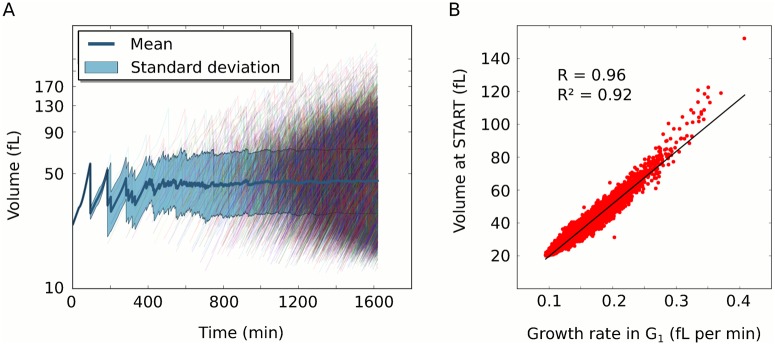
Population size homeostasis through growth rate dependent size control in G_1_ (Model-1). A population of fast growing cells (glucose, final number: 10.000) was simulated with Model-1 for analysis. (A) Population size homeostasis emerges through the dynamics of all individual cells (thin coloured lines). After division, cells restart at lower volumes. Since always two cells result from one dividing cell, thin lines become denser over time. The thick dark blue line indicates the average cell size in the population and the light-blue area the range of one standard deviation (SD). Despite the diverse behaviour of the individual trajectories, average and SD stabilise quickly when the population grows starting from one individual at time 0 minutes. (B) Volume at START correlates with the growth rate in G_1_ for individual cells in the model. Growth rate in G_1_ was calculated as the difference between volume at START and the birth volume divided by the duration of G_1_. Black line indicates a least-squares regression with correlation coefficient (R) and coefficient of determination (R^2^).

To test the effect of *mCLB2* localization on size control, we implemented two model versions. In Model-1, *mCLB* is uniformly distributed in the cell. It is translated in the entire cell. In this model, the biosynthetic capacity of the whole cell is integrated at the G_2_/M transition (G_2_ size control of the entire cell). In Model-2, the mRNA is translated exclusively in the newly forming bud mimicking the effect of mRNA localization. This model integrates only the biosynthetic capacity of the bud in G_2_ (G_2_ bud size control). Regardless of the specific G_2_ size control mechanisms, in both models, the productive capacity of the cell is measured in G_1_ through translation of *mCLN* (G_1_ size control), the primary phase for size control in budding yeast [1, 45]. The two models differ in a single equation (Table 1). *In silico* cultures generated with Model-1 and Model-2 show size homeostasis on the population level (Figs 2A and S1). Moreover, in both models there is a strong dependence of the cell volume at START on the individual growth rate in G_1_ phase (Figs 2B and S1–S3), as observed experimentally [15]. Thus, in accordance with experiments, both models exert growth rate dependent size control primarily in G_1_, regardless of the *mCLB2* localization in G_2_ [1, 15, 45].

**Table 1:** Model equations.

1.	$\frac{\text{d}}{\text{d}t}mCLN=-k_{d}\cdot f(mCLN,t)$
2.	$\frac{\text{d}}{\text{d}t}\text{Cln}=k_{p1}\cdot mCLN\cdot B^{R}\cdot \frac{A(t)}{V(t)}-k_{d}\cdot \text{Cln}$
3.	$\frac{\text{d}}{\text{d}t}mCLB=-k_{d}\cdot f(mCLB,t)$
4.1	$\frac{\text{d}}{\text{d}t}\text{Clb}=k_{p2}\cdot mCLB\cdot B^{R}\cdot \frac{A(t)}{V(t)}-k_{d}\cdot \text{Clb}$
4.2	$\frac{\text{d}}{\text{d}t}\text{Clb}=k_{p2}\cdot mCLB\cdot B^{R}\cdot \frac{A(t)}{V(t)}\cdot \frac{V^{d}(t)}{V(t)}-k_{d}\cdot \text{Clb}$
5.	$\frac{\text{d}}{\text{d}t}B^{R}=growth\cdot (\frac{k_{R}}{k_{R}+k_{Am}+k_{Ad}})\cdot mB^{R}\cdot B^{R}\cdot \frac{A(t)}{V(t)}$
6.	$\frac{\text{d}}{\text{d}t}B^{Am}=growth\cdot (\frac{k_{Am}}{k_{R}+k_{Am}+k_{Ad}})\cdot mB^{A}\cdot B^{R}\cdot \frac{A(t)}{V(t)}$
7.	$\frac{\text{d}}{\text{d}t}B^{Ad}=growth\cdot (\frac{k_{Ad}}{k_{R}+k_{Am}+k_{Ad}})\cdot mB^{A}\cdot B^{R}\cdot \frac{A(t)}{V(t)}$
8.	$\frac{\text{d}}{\text{d}t}mB^{R}=0$
9.	$\frac{\text{d}}{\text{d}t}mB^{A}=0$
10.	$f(mRNA,t)=\{\begin{array}{cc} mRNA(t_{i})+randint(0,1\to P_{x}) & \text{if}\;t=t_{i}\;\text{for}\;x\in \{mCLN,mCLB\}\\ mRNA(t) & \text{otherwise}\; \end{array}$
11.	△*A* ^*m*^ ≡ △*B* ^*Am*^
12.	△*A* ^*d*^ ≡ △*B* ^*Ad*^
13.	*A*(*t*) = *A* ^*m*^(*t*)+*A* ^*d*^(*t*)
14.	*V* ^*x*^(*t*) ∝ (*A* ^*x*^(*t*))^3/2^ for *x* ∈ {*m*, *d*}
15.	*V*(*t*) = *V* ^*m*^(*t*)+*V* ^*d*^(*t*)

Equation 4.1 is implemented in Model-1, equation 4.2 is implemented in Model-2.

### Clb2 constitutes a growth rate dependent sizer candidate in G_2_

**Fig 3:**
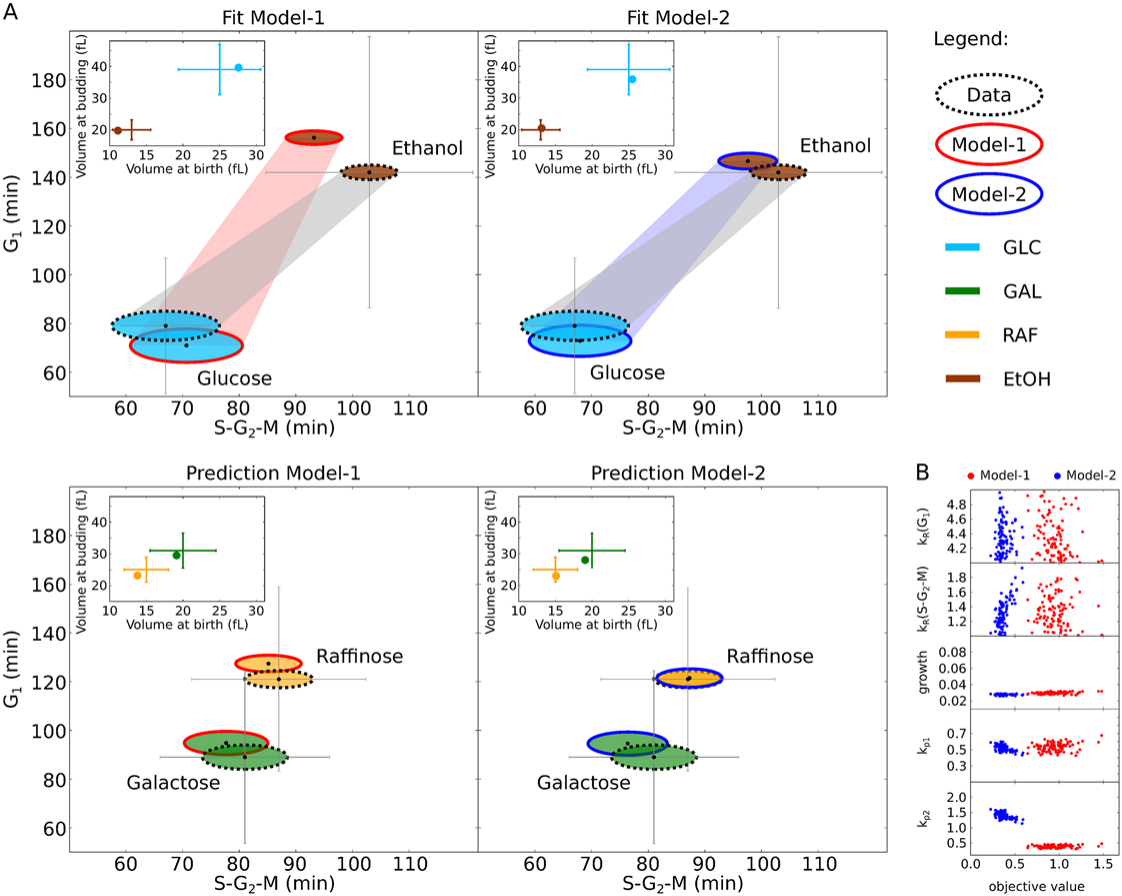
The Models reproduce diverse nutritional conditions. (A) Best fit of Model-1 (left) and Model-2 (right) to cell size and proliferation time data is shown. Proliferation time is indicated as G_1_ and S-G_2_-M duration (black dots, grey error bars); corresponding cell size is represented by ovals (oval height: size at birth; width: size at budding). The nutritional conditions glucose (GLC—light blue) and ethanol (EtOH—brown) were used for fitting (top); galactose (GAL—green) and raffinose (RAF—yellow) for model validation (bottom). For clarity, inset figures show volume at birth and at budding again, the values correspond to oval sizes (exp. data: error bars, simulation: dots). A shadow in the top panel indicates how growth and proliferation change over conditions (exp. data: grey, Model-1: light red, Model-2: light blue). All experimental data is taken from [15]. (B) Parameter distributions over objective values (Materials and Methods Eq 4) of 100 independent parameter estimations started from uniformly distributed parameter values within boundaries (y-axis ranges correspond to boundaries, see also Table 2) for Model-1 (red) and Model-2 (blue).

For the models to become reliable mathematical tools to investigate the coupling of growth and division they were fitted independently to complex experimental growth and proliferation data (Fig 3) [15]. The data is available for cells grown on glucose, galactose, raffinose and ethanol [15]. In the models, simulation of cell growth on different carbon sources can be achieved by modulating the parameter *growth* (Table 2). The parameter *growth* scales the biomass formation and represents the availability of nutrients to the system (Table 1). A decrease in *growth* leads to a reduction of cell size and a prolongation of the cell cycle [32]. We used the data for glucose and ethanol for parameter estimation and the data for galactose and raffinose for validation of the models (Fig 3A). The antagonistic trend within the data (‘fast growth → short cell cycle → large cells’ versus ‘slow growth → long cell cycle → small cells’, shown in Fig 3A as a grey shadow) enables to constrain most of the model parameters (Fig 3B, Materials and Methods). Although parameters show correlations (S4 Fig), within the parameters boundaries the fits converge into a global minimum (Figs 3B, S5 and S6). Both model variants predict cell size at birth, at budding, and duration of G_1_ and S-G_2_-M for all conditions with high accuracy. To test which model is more suitable to describe the given data, we ranked them using the Akaike Information Criterion (AIC) [46]. Ranking yields that Model-2 fits the data better than Model-1, although both versions use the same number of parameters (S1 Table). The worst fit of Model-2 is still better than the best fit of Model-1 (Fig 3B). This indicates that the mechanism of compartmentalization (bud-localised *mCLB*) renders the system more capable to describe (glucose and ethanol) and predict (galactose and raffinose) the data [15].

**Table 2:** Model parameters.

parameter	Model-1	Model-2	Boundaries	specification
*growth*	0.028	0.029	[0.01, 0.1]	growth condition
*k*_*p*1_	0.452	0.589	[0.1, 1.0]	Cln production ((min⋅mol)^−1^)
*k*_*p*2_	0.342	1.606	[0.1, 2.5]	Clb production ((min⋅mol)^−1^)
*k*_*R*_(G_1_)	4.92	4.098	[4.0, 5.0]	biosynthetic capacity growth in G_1_
*k*_*R*_(S-G_2_-M)	1.495	1.04	[1.0, 2.0]	biosynthetic capacity growth in S-G_2_-M
*k*_*Am*_(G_1_)	1			mother cell growth in G_1_
*k*_*Am*_(S-G_2_-M)	0			mother cell growth in S-G_2_-M
*k*_*Ad*_(G_1_)	0			bud growth in G_1_
*k*_*Ad*_(S-G_2_-M)	1			bud growth in S-G_2_-M
*k*_*d*_	0.1			degradation of proteins and mRNA (min^−1^)
*P*_*x*_	0.4			probability of mRNA burst (min^−1^)
*threshold*	150			cyclins for critical kinase activity (molecule number)
*S*-*phase*	25			duration of S-phase (min)
*M*-*phase*	5			duration of mitosis (min)

The parameter values for the best fit of Model-1 and Model-2 are displayed. The parameters *growth*, *k*_*p*1_, *k*_*p*2_, *k*_*R*_(G_1_) and *k*_*R*_(S-G_2_-M) differ for the two model versions, whereas the other parameters have the same value in both models. Parameter boundaries are shown for estimated parameters. Units of parameters without specification are dimensionless.

Experimental data clearly show that S-G_2_-M duration is not constant between different media [13–15]. Since S and M phase are constant, a G_2_ regulator is needed to account for the adaptation of G_2_ duration in response to nutritional status *in vivo* [13]. Since both models reproduce the experimental data for glucose, galactose, raffinose and ethanol (Fig 3A), we conclude that, regardless of compartmentalization, translation of *mCLB* is a good candidate. The proposed mechanism also leads to correlation between the volume of the bud at division and the individual growth rate in the budded phase as well as the S-G_2_-M duration (S7–S9 Figs). Thus, Clb2 constitutes a growth rate dependent sizer candidate in G_2_.

### *mCLB* localization reduces noise at mitotic entry to stabilise cell size

**Fig 4:**
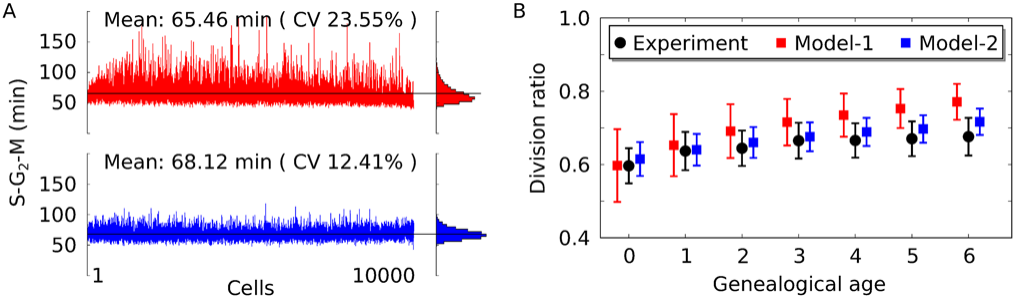
A growth rate dependent bud sizer tunes mitotic entry to maintain size ratios at division. (A) Deviation around the mean duration of the budded phase (S-G_2_-M) is shown for fast growing cells (glucose) simulated with Model-1 (red) and Model-2 (blue). The mean of the data (black line) and the coefficient of variation (CV) are also shown. (B) Volume fraction of cell and bud at division is shown as a function of replicative age for data from experiments [41] (black dots) and from simulations with Model-1 (red squares) and Model-2 (blue squares).

To further distinguish the models, we analysed the effect of mRNA localization on other systems level properties beyond average size and cell cycle phase duration. It has been reported that a growth rate dependent sizer can prevent large fluctuation of G_1_ length to reduce the generation time on the population level [15]. We find that compartmentalization of Clb translation (Model-2) reduces fluctuations of G_2_ length (Fig 4A). Interestingly, this does not lead to a reduction in generation time on the population level (S10 and S11 Figs). The higher noise at mitotic entry, inherent in Model-1, propagates directly to cell division ratios (Fig 4B), where cells that spend too much or little time in S-G_2_-M produce abnormally large or small buds, respectively. In comparison with experiments, Model-1 predicts a too high variability in division ratios for the first five generations of the population, i.e. for more than 95% of the cells in the culture [41]. In contrast to Model-1, the predictions of division ratios from Model-2 are accurate for young and old cells (Fig 4B). This is even more pronounced for slow growing cells (S12 Fig). Thus, a bud sizer can tune mitotic entry to reduce noise and maintain division ratios over generations.

**Fig 5:**
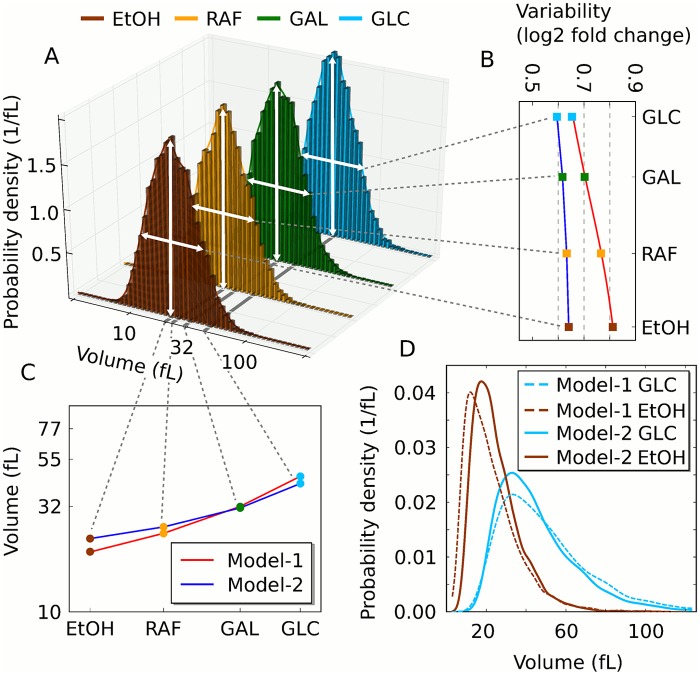
Cell size statistics. (A) Cell size distributions are shown for *in silico* cultures simulated with Model-2 in glucose (GLC—light blue), galactose (GAL—green), raffinose (RAF—yellow) and ethanol (EtOH—brown). (B-C) Relative variability in cell size (B) and average cell size (C) is shown for *in silico* cultures obtained from simulations with Model-1 (red) and Model-2 (blue) in same conditions as in (A). (D) Cell size distributions density estimates of Model-1 (dashed lines) and Model-2 (solid lines) on a linear scale. Cell size is log-normally distributed [32], hence size average in (B) and relative variability in (C) are computed on log-values.

It is well known that cells grown on rich media grow faster and are larger compared to cells grown on poor media [47]. Previously, we analysed cell size distributions of cells grown on rich and poor media and found that, in contrast to average cell size, the relative variability in cell size does not change [32]. A model that allowed size control exclusively in G_1_ (constant S-G_2_-M) could not explain this [32]. Model and data could only be reconciled when we adapted S-G_2_-M duration to growth conditions. Thus, we hypothesised that, for a robust setting of average cell size and variability within the culture, S-G_2_-M duration must show some form of adaptation to growth conditions. We have seen that Model-1 and Model-2 are able to reproduce and predict the adaptation of S-G_2_-M duration in response to different conditions (Fig 3). To test whether both versions stably reproduce an increase in average cell size while keeping the relative variability constant, we analysed their behavior with respect to cell size statistics (Fig 5). It is apparent that both versions show the expected increase in average cell size, but that relative variability is increased in Model-1. Model-1 shows an increase in relative variability of roughly 25% over different conditions (glucose ∼ 0.65, ethanol ∼ 0.81), whereas Model-2 of only 7% (glucose ∼ 0.60, ethanol ∼ 0.64). This indicates that a growth rate dependent G_2_ bud sizer stabilises the relative variability in cell size observed in yeast populations [32].

### *mCLB* localization is required to equilibrate S-G_2_-M duration between generations

**Fig 6:**
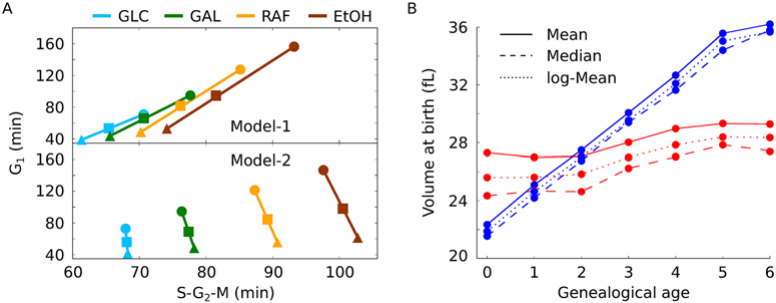
A bud sizer is required to equilibrate the budded phase of mothers and daughters and leads to larger daughters from older mothers. (A) G_1_ and S-G_2_-M durations are displayed for populations simulated with Model-1 (top) and Model-2 (bottom) in glucose (GLC—light blue), galactose (GAL—green), raffinose (RAF—yellow) and ethanol (EtOH—brown). Durations are averaged for daughters (circles), mothers (triangles) or the entire population (squares). Lines show the difference between mothers and daughters. (B) The size of new-born cells is shown as a function of the replicative age of the mother for Model-1 (red) and Model-2 (blue). Shown are the mean of the culture, the median and the mean of log-transformed values.

Both model versions show adaptation of S-G_2_-M duration to growth conditions (Fig 3). Hence, according to our hypothesis (adaptation of S-G_2_-M stabilises the variability—see last paragraph), both models should in theory be able to stabilise the variability [32]. However, only Model-2 is able to limit the size variability (Fig 5). To explain this, we analysed the apparent S-G_2_-M adaptation in both models. The difference between Model-1 and Model-2 in G_1_ and S-G_2_-M duration seems minor under most conditions (Fig 3). However, the difference between the models becomes striking when inspecting the time that daughter and mother cells spend in G_1_ and S-G_2_-M separately (Fig 6A). Apparently, in Model-1 mother and daughter lines diverge, whereas in Model-2 they do not. In Model-1, time in G_1_ is different for mothers and daughters, as expected [48]. This difference is larger for cells grown on ethanol than for cells grown on glucose, also as expected [14]. In Model-1, the time in S-G_2_-M differs for mothers and daughters as well, which is in clear contrast to experimental evidence [13, 14]. Single cell data has shown in detail that there is little difference for time in S-G_2_-M between daughters and mothers (Table 3) [14]. Model-2 also displays the expected differential G_1_ duration between mother and daughter cells, which again decreases with nutrient quality, as expected (Fig 6A) [48]. In contrast to Model-1, the S-G_2_-M duration of mothers and daughters in Model-2 is more equilibrated, very similar to experimental findings (Table 3). Yet, there is a trend in Model-2 that for slow growing cells budded phase is longer for mother than daughter cells. A consequence of the equilibrated budded phase within the population (Model-2), is that the volume of new born daughter cells increases with the age of the corresponding mother (Fig 6B). As a result, daughters of old mothers are considerably larger than daughters from young mothers in Model-2. This observation has also been made *in vivo* [49], suggesting that a mechanism exists that controls the size of the bud, rather than absolute cell size, in G_2_.

**Table 3:** Duration of budded phase.

		Model-1	Model-2	Experiment
glucose	mother	61	68	72
	daughter	71	68	76
	difference	10	0	4
ethanol	mother	74	103	101
	daughter	93	98	106
	difference	19	-5	5

Time in S-G_2_-M (minutes) is shown for *in silico* (Model-1 and Model-2) and *in vivo* data (Experiment) for cells grown on glucose and ethanol. The difference between mother and daughter value is shown as well. Experimental data is taken from [14].

### A bud sizer, not a whole-cell sizer, is required in G_2_ to predict the *CLN3* overexpression *whi* mutant phenotype

**Fig 7:**
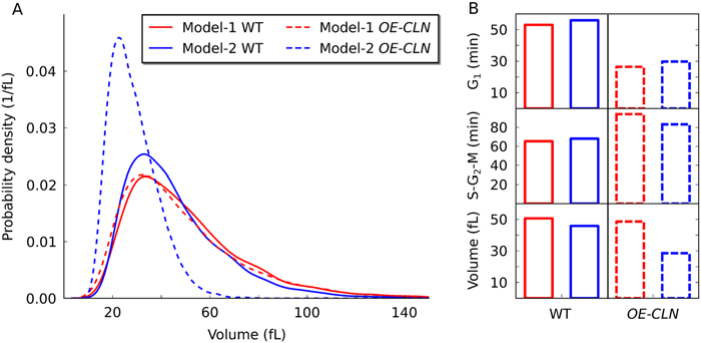
A bud sizer is required to predict the *CLN* overexpression mutant. (A) Cell size distributions are shown for the fast growing wild type (WT, solid lines) and *CLN* over producing mutant (*OE-CLN*, dashed lines) simulated with Model-1 (red) and Model-2 (blue), corresponding to a *CLN3* overexpression mutant *in vivo*. (B) Average length of G_1_, S-G_2_-M and average cell volume are shown for the wild type (solid bars) and *OE-CLN* cells (dashed bars) simulated with Model-1 (red) and Model-2 (blue).

To further compare the predictive power of the models, we simulated a scenario similar to over producing *CLN3*. We enforced a doubling of *CLN* expression in the models (referred to as *OE-CLN*). Both models react to the overproduction of *CLN* with a decrease in G_1_ duration and a compensatory increase of the budded phase duration (S-G_2_-M; Fig 7). In agreement with experimental observations, average generation times are only slightly (Model-2) or not at all (Model-1) reduced by the mutation (S10 and S13 Figs) [33, 34]. However, the difference between cell cycle durations of mothers and daughters is reduced in the mutant (S13 and S14 Figs). Yet, only Model-2 shows a the reduction in cell size in response to *OE-CLN* (Fig 7). Model-1, with the whole-cell G_2_ sizer, fails to predict the small cell size phenotype that is typical for *CLN3* overexpressing cells [33, 34]. This shows that a bud sizer (Model-2) is required to predict the *CLN3* overexpressing strain’s *whi* mutant phenotype.

## Discussion

Here, we present a mechanistic single cell growth model that is able to predict cell growth and division timing in budding yeast populations. There are very few models that are able to (i) show and explain size homeostasis, (ii) offer mechanistic insight into the cellular machinery governing growth and division, and (iii) that are still comprehensible and manageable [32, 50]. Our model is designed to omit all details that are not absolutely required to reproduce the coordination of growth and proliferation. Using the model we show that a G_2_ bud sizer mechanism is required in addition to a G_1_ sizer in order to (i) better fit and predict population size and timing data for different nutritional conditions (Figs 3, 4B and 5, Table 3), (ii) offer a functional explanation for the experimentally observed *mCLB2* transport into the bud [35], (iii) reduce the noise at mitotic entry (Figs 4 and S12) and (iv) render the model capable of predicting the phenotype of a *CLN3* overproducing strain (Fig 7). Thus, our results indicate that a bud sizer mechanism could operate at the G_2_/M boundary *in vivo* [10].

We use the model to show that biomass dependent accumulation of cyclins to a threshold results in size homeostasis on the population level and growth rate dependent size adaptation in G_1_ (Fig 2), as seen *in vivo* [15]. Thus, our results are in accordance with the view that G_1_ is the primary phase for size control in budding yeast [1, 45]. It was shown that the START network sets the cell size as a function of the growth rate [15]. We observe a similar behaviour in our model, meaning that we implement a growth rate dependent sizer through a minimal version of the START network. The model proposes that the underlying network developed from a single CDK-cyclin pair that later differentiated between G_1_ and G_2_ phase. Consistently, cells can be driven through the cell division cycle by artificial expression of a single CDK-cyclin fusion protein [43]. While we observe a stronger correlation between the cell volume at START and the individual growth rate than seen in experiments (Fig 2B) [15], this can be explained by the simplicity of the model and the lack of feedbacks and other regulatory mechanisms [23, 26, 51, 52]. Taken together, the model predicts (a) that already a single G_1_ cyclin suffices for size control in G_1_ and (b) that monitoring biosynthetic capacity through production of a critical unstable cyclin is a growth rate dependent sizer.

In previous work, we found that a growth model with size control operating exclusively during G_1_ is not able to fully reproduce data of cell sizes and proliferation times measured for different conditions [32]. Also, recent experimental evidence strongly supports the existence of a size-regulating mechanism in the budded phase [10]. Accordingly, we tested models with additional size control at the G_2_/M transition for the entire cell (Model-1) or only for the bud (Model-2). The G_2_ size control is implemented analogously to the G_1_ sizer, through biosynthetic capacity dependent production of a critical cell cycle regulator. The models establish that a G_2_ size control point, but not necessarily bud size control, is required to reproduce the experimentally observed adaptation of the budded phase in poor growth media (Fig 3) and in the *CLN3* overexpression mutant (Fig 7) [13–15, 33, 34]. Hence, both models argue in favor of the existence of a sizer mechanism in G_2_. Yet, simply regulating G_2_ length in response to growth conditions does not seem to be the end of the story. Conceptually, the reduction of G_1_ and the compensatory adaptation of the budded phase, inherent in both models, is not sufficient to explain the small size phenotype of the *CLN3* overproducer (Figs 7 and S14).

The prolongation of the budded phase has different reasons in Model-1 and Model-2. In Model-1, cells pass START quickly with a reduced biosynthetic capacity (and size) as a direct consequence of the *CLN* overexpression. Since Model-1 is implemented to integrate the whole cell’s biosynthetic capacity at mitotic entry, cells compensate by extending S-G_2_ to build up sufficient productive power to enter mitosis. The requirement to enter mitosis here equals the one in the wild type so that, in Model-1, *CLN* overexpressing cells are very similar in size compared with wild type cells. A reduction in growth rate, due to early passage through START is compensated late in the cell cycle when enough biosynthetic capacity has built up. Consequently, cells overexpressing *CLN* in Model-1 tend to have larger buds leading to larger cells at birth (S14 Fig). In contrast, the prolongation of the budded phase seen in Model-2 is due to a general reduction in growth rate, which is again the consequence of the early passage through START. In Model-2, the biosynthetic capacity of the bud is monitored, which accumulates slower because of the generally reduced growth rate. However, in Model-2, a cell can enter mitosis as long as the bud fulfills minimal requirements, even if the cell in total is smaller and less productive. This is in accordance with the fact that entry into mitosis is correlated with the size of the bud, but not with the size of the mother cell [17]. Thus, only the bud sizer and not a whole cell sizer concept at mitotic entry is able to accommodate the small size phenotype of the *CLN3* overproducer and can thus reconcile model and experimental observations [33, 34].

We found that the budded phase is slightly longer in mothers than in daughters for slow growing cells in Model-2 (Fig 6A). While such an effect might be too small to be detected in experiments, it is more likely that the model oversimplifies at this point: The assumption that 100% of the *CLB2* transcript is transported to and exclusively translated in the bud *in vivo* could be too strong. Indeed, it is difficult to assess the exact number of bud-localised transcript (100% in the model and ≥ 90% as reported experimentally [35]). Predictions of Model-1 show the effect of non-localization, i.e. shorter budded phase in mothers. This is indicative that allowing some translation of the cyclin in the mother (e.g. 10%) can eradicate the difference.

We predict bud-localised *mCLB2* to be an essential part of the growth and division coupling. Obviously, we cannot be sure that we have implemented the true biological mechanism in our model, but we show here that our minimal mechanism displays many characteristic properties of the system. It is tempting to speculate that the G_2_ bud sizer is of similar design as the one operating in G_1_, since it seems easier to duplicate a working mechanism than to invent two distinct, yet functionally equivalent ones. Still, we acknowledge that there are other hypotheses on how cyclin synthesis is related to growth rate [30, 31, 40, 53]. Most likely, the *in vivo* situation is the complex result of different interacting molecular effects. Nonetheless, the mechanism proposed here suffices to explain most of the data, and—unlike many other hypotheses that rely on a specific molecular mechanism in the G_1_ phase—the mechanism proposed here is generic and not phase specific. In light of this, passive accumulation of CDK regulators (Cln3 in G_1_ and Clb2 in G_2_) are promising candidates [8]. The old concept of the unstable regulator is seductively simple and elegant [25]. By making the underlying size control mechanism depending on the critical CDK activity induced by an exchangeable regulator (cyclins can substitute for one another) one could explain why none of the components, neither concerning the START network nor the morphogenesis checkpoint, are absolutely indispensable [43, 54, 55].

The importance of the mRNA localisation, as we highlight it here, can possibly be tested experimentally. The polarised localisation of *mCLB2* could be perturbed by disruption of the sequence in *mCLB2* required for transport (if identified), or by deletion of the *MYO4* gene that encodes the type V myosin motor responsible for bud-localisation of mRNA [56]. Such perturbations would revert the phenotype from Model-2 to Model-1. To discriminate the two experimentally, the *MYO4* deletion would need to be combined with a *CLN3* overexpression. The model predicts the loss of the *whi* phenotype in this double mutant, which should be detectable in an experimental setting (Fig 7). However, this experiment involves two major genetic perturbations and the outcome may not be as clean as the *in silico* experiment.

Seeing that *mCLB2* is localised to the bud of the cell and during S-G_2_-M mainly the bud grows, it is likely that a G_2_ sizer actually has an effect on bud size rather than absolute cell size *in vivo* [35]. Also, while measuring the biosynthetic capacity of a cell in G_1_ works well to ensure that it is strong enough for duplication, measuring the capacity again in G_2_ (cell and bud—Model-1) seems futile and less accurate. It seems rather more useful to measure the capacity only of the bud (Model-2), ensuring that the new descendant is strong enough for independent life. From a population’s perspective, it is reasonable to control the offspring’s size at least as strictly as individual cell size, maybe even with the same mechanism.

**Fig 8:**
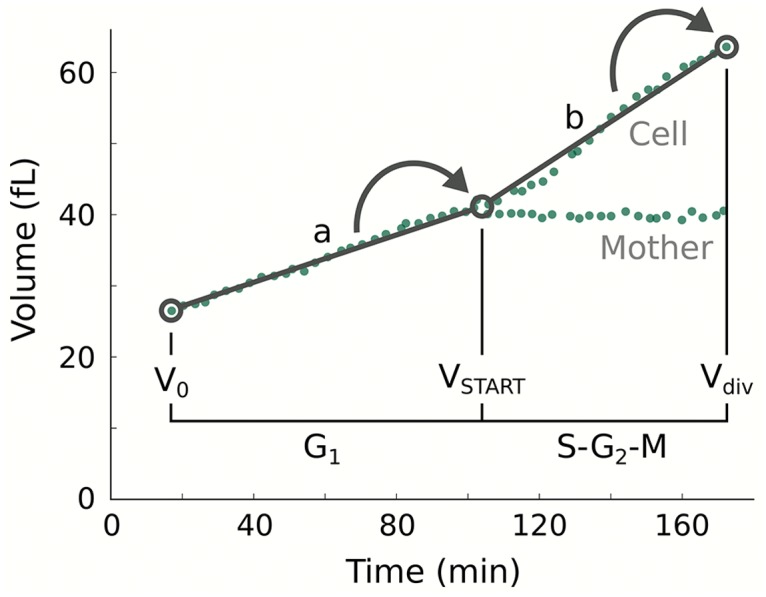
Unifying model for G_1_ and G_2_ cell size control. Cell size at the G_1_/S and the G_2_/M transitions is controlled in a growth rate dependent fashion with a functionally equivalent mechanism. Cell size at START (V_START_) is a function of the growth rate in G_1_ (a), as recently shown [15]. Cell size at division (V_div_) is a function of the growth rate in G_2_ (b), as proposed here. Experimental data (green dots) is taken from [40].

Taken together, we propose a model where cell size in budding yeast is controlled at the cell cycle junctions G_1_/S and G_2_/M in a growth rate dependent fashion (Fig 8). Our results suggest that the simple mechanism we employ here at both transitions is sufficient. Moreover, the model works without setting of a ‘critical cell size’. Considering the growth medium dependent nature of the ‘critical cell size’ itself, this strongly advocates an interpretation of cell size as an emergent property of the coupling between growth and division, rather than a regulatory parameter. In accordance, cell size at START would be a function of the growth rate in G_1_. Strong evidence in this direction recently emerged [15]. We propose here that bud size at division is a function of the growth rate in S-G_2_-M, meaning that there is a common size control theme in the two growth phases of the cell division cycle. This can explain the prolongation of both phases, G_1_ and G_2_, in response to poor nutrients and the small size phenotype seen for *CLN3* overexpression [14, 15, 33, 34]. In conclusion, we present a cell growth model, which unifies integration of growth and division in the G_1_ and G_2_ phases of the cell division cycle to accurately reproduce and predict cell size at birth and at budding, as well as timing of the cell cycle phases over four different nutritional conditions for budding yeast.

## Materials and Methods

### The model

The model is an extension of a minimal eukaryotic cell model [32]. It is implemented with ordinary differential equations, stochastic functions and algebraic equations (Table 1). Two species were added (*mCLB* & Clb), such that Cln drives the cell cycle in G_1_ phase and Clb in G_2_. Hence, transcription of *CLN* is restricted to G_1_ and of *CLB* to G_2_. By default, the model links metabolism to progression through the cell cycle via biomass dependent accumulation of the two regulatory proteins (G_1_ cyclin Cln and G_2_ cyclin Clb), whereas the synthesis phase (S-phase) and the Mitose (M-phase) simply delay cell cycle progression (see Table 2). The cell cycle of the model has four transitions, corresponding to the eukaryotic phase transitions (G_1_/S, S/G_2_, G_2_/M, M/G_1_). The model equations governing the dynamics are displayed in Table 1.

The model rests on a set of explicit assumptions: namely, that nutrient supply is defined by uptake, which is proportional to cell area; that transcription is stochastic and that nutrient incorporation into biomass relies on the biosynthetic capacity of the cell. Thus, production reactions are dependent on precursors and the internal biomass. The efficiency of nutrient incorporation is inversely scaled with volume to reflect dilution. Furthermore, that the total area of the cell is the sum of the area of the mother and the bud. Correspondingly, we calculate the total volume of the cell as the sum of mother and bud volume. Mothers and buds are approximated as separate spheres, thus *V* ∝ *A* ^3/2^. As a cell grows, the ratio of the area to the volume shifts, since the area expands slower than the volume. Given our above stated assumption about the influence of nutrient supply (area) and dilution (volume), it follows that the decrease of the area-to-volume-ratio places an increasing constraint on the cellular biosynthetic capacity slowing down growth [32]. The idea that the surface area-to-volume-ratio plays an important role in connecting the cell growth to the cell division cycle was also explored by others [50, 57]. In our model, cells may allocate their resources according to cell cycle stage, which means that resources can be used to form structural or internal biomass in different proportions in different cell cycle stages. Specifically, we assume that the increase of the biosynthetic capacity is strong in G_1_ (heavier allocation of resources to internal biomass) and less so during S-G_2_-M [58, 59]. The structural biomass can furthermore be distributed to either the area of the mother cell or the area of the bud. There is no bud growth during G_1_ and we assume that there is only bud growth during S-G_2_-M [15, 40]. Finally, we simplify phase transitions to a threshold for nuclear kinase activity, assuming zero order ultra-sensitivity [60].

The model itself is a single cell model that can grow and divide (S1 File). To model entire asynchronously growing cell cultures, however, we developed an algorithm to simultaneously simulate a growing ensemble of the single cell models [32]. An executable and editable version of the algorithm implemented in python is added in the Supporting Information (S2 File). During the simulation, a cell grows during G_1_, then it grows a bud during S-G_2_-M. At division, the bud is detached from the mother cell. The mother cell starts a new cell cycle and, additionally, a new cell instance is created according to the size of the detached bud. All soluble components are split at division between the two cells according to the volume ratio of the mother and the bud. In this fashion, two distinct cells are created from one cell. The two cells differ in starting conditions, e.g. cell size or biosynthetic capacity, which lead to differential growth and proliferation properties for the individual cell, e.g. growth rate or time spent in G_1_. Simulation of cell cultures in different nutrients is implemented through the parameter *growth*, which is used to scale the biomass formation to control the nutrient availability of the system (Tables 1 and 2). In both models we use the constraint that *growth*_*ethanol*_ = *growth*_*glucose*_⋅0.5. Similar relations are used to simulate galactose (*growth*_*galactose*_ = *growth*_*glucose*_⋅0.77) and raffinose (*growth*_*raffinose*_ = *growth*_*glucose*_⋅0.6).

We implemented two different versions of the model. Note that the only difference between the two is equation 4. Model-1 uses equation 4.1 and Model-2 is implemented with equation 4.2. Model version 2 is based on the fact that *mCLB2* is actively transported into the nascent bud in *S. cerevisiae* [35]. However, since the model does not include spatial displacement of components, we employed a work-around to implement the consequence of the transport. Assuming that the important function of *mCLB2* transport into the bud is to localise translation to this sub compartment, we allowed only the fraction of ribosomes in the bud to translate the *CLB* mRNA. This is why, in equation 4.2, the metabolic capacity (*B* ^*R*^) is scaled with the term *V* ^*d*^(*t*)/*V*(*t*), assuming well-stirred conditions.

### Parameter estimation

Model-1 and Model-2 contain 14 parameters each, five of which we estimated using a maximum likelihood approach (see Table 2). Both model versions were fitted independently to experimental data from Aldea and colleagues who analysed asynchronously growing daughter cells using time-lapse microscopy [15]. We used two different though related types of their data to constrain our parameters: (i) time at START (T1), budding (T2) and division (T3); (ii) volume at birth (V0) and budding (Vbud). Aldea and colleagues provide the mean value (*μ*) and coefficient of variation (cvar in %) for both types of data for daughter cells grown on four different carbon sources (glucose, galactose, raffinose and ethanol). Here, we used the data for glucose and ethanol for parameter estimation. We recalculated the standard deviation (*σ*) from the cvar and the mean, such that *σ* = *μ*⋅*cvar*/100. The data and the model fits are shown in Fig 3.

In the model we do not distinguish between START transition (T1) and budding (T2) but consider that as soon as the threshold is crossed, the *in silico* cells enter S-phase and G_1_ is finished. As such, for this special case, we relate the model and the data as follows. Time in G_1_ (TG1) from the model equals the experimental time at START (T1) plus the time from START to budding (T2)

$$\begin{array}{c} \mathrm{TG}1=T1+T2. \end{array}$$ (1)

Now the mean data point is the sum of T1 and T2 with two stdevs *σ*_*T*1_ and *σ*_*T*2_, respectively. Neglecting correlations of T1 and T2, the formula for propagation of uncertainties provides an approximation of the combined error

$$\begin{array}{c} \sigma_{f}=\sqrt{{\left(\frac{\partial f}{\partial x_{1}}\right)}^{2}\sigma_{x_{1}}^{2}+{\left(\frac{\partial f}{\partial x_{2}}\right)}^{2}\sigma_{x_{2}}^{2}} \end{array}$$ (2)

where *f* = *x*_1_+*x*_2_ (see Eq 1) [61]. This simplifies to yield the combined error for TG1

$$\begin{array}{c} \sigma_{\mathrm{TG}1}=\sigma_{\left(T1+T2\right)}=\sqrt{\sigma_{T1}^{2}+\sigma_{T2}^{2}}. \end{array}$$ (3)

As objective function for the fitting of parameters we chose the weighted sum of squared residuals (wRSS) given by the measured values *x* and the simulated values $\hat{x}$, such that

$$\begin{array}{c} \mathrm{wRSS}=\sum {\left(\frac{x-\hat{x}}{\sigma }\right)}^{2}. \end{array}$$ (4)

Minimization of Eq 4 is equivalent to maximizing the log-likelihood given by

$$\begin{array}{c} ln\left(L\left(p\right)\right)=-\frac{m}{2}ln\left(2\pi \right)-\sum ln\left(\sigma \right)-\frac{1}{2}\sum {\left(\frac{x-\hat{x}}{\sigma }\right)}^{2}. \end{array}$$ (5)

It is important to stress at this point that the experimental data was generated analysing only daughter cells [15]. Accordingly, only *in silico* daughter cells we used to calculate the appropriate data $\hat{x}$.

Model-1 and Model-2 were fitted independently using a custom evolutionary parameter estimation algorithm (S1 Text). The algorithm is suitable for fitting population data and has been used for all parameter estimation tasks. The algorithm allows specification of parameter boundaries for the estimation (parameter boundaries are shown in Table 2). The estimation was performed for 100 uniformly distributed initial values (in the range of the parameter boundaries) for the parameters which enabled us to derive the parameter correlations (S4 Fig).

We estimate five out of 14 parameters because to a certain degree we anticipated parameter correlations, over-fitting or under-determination of parameters since the nature of the data (population averaged) and the parameters (single cell) are distinct. There are correlations in the parameters that the data cannot account for. For example, the parameters for Cln protein production *k*_*p*1_, degradation *k*_*d*_, probability of *mCLN* synthesis *P*_*mCLN*_ and the threshold value (Table 2) together influence the duration of G_1_ phase. Their combined effect determines the final duration. Since the given data concerns the duration, we cannot hope to estimate the true value of the four influential parameters but only their combined effect. This is why we set three out of four to a fixed value and estimated the fourth, such that the global effect matches the data. We can thus not report unique kinetic parameters for protein production and degradation but values that are useful in combination to describe the global process that determines G_1_ duration.

Accordingly, our approach enables us to describe the global effect and also distinguish different model versions. To find the model version that would best approximate reality given the data and the number of parameters we employed the Akaike Information Criterion (AIC) to rank the models [46]. The AIC establishes a relationship between the maximum likelihood and the Kullback-Leibler information, which is a measure for the information lost when approximating reality with a model [62]. The AIC was computed as

$$\begin{array}{c} AIC=-2(ln(L(p)))+2K \end{array}$$ (6)

with *K* being the number of estimated parameters in the model. Model statistics with respect to the objective function, the log-likelihood and the AIC are summarised in S1 Table.

## Supporting Information

S1 Fig Population size homeostasis through growth rate dependent size control in G_1_ (Model-2). A population of fast growing cells (glucose, final number: 10.000) was simulated with Model-2 for analysis. (A) Population size homeostasis emerges through the dynamics of all individual cells (thin coloured lines). After division cells restart at lower volumes. Since always two cells result from one dividing cell, thin lines become denser over time. The thick dark blue line indicates the average cell size in the population and the light-blue area the range of one standard deviation (SD). Despite the diverse behaviour of the individual trajectories, average and SD stabilise quickly when the population grows starting from one individual at time 0 minutes. (B) Volume at START correlates with the growth rate in G_1_ for individual cells in the model. Growth rate in G_1_ was calculated as the difference between volume at START and the birth volume divided by the duration of G_1_. Black line indicates a least-squares regression with correlation coefficient (R) and coefficient of determination (R^2^).
(PDF)

S2 Fig Correlations of G_1_ duration, the growth rate in G_1_ and volume at START in Model-1. A fast growing (glucose) culture was simulated with Model-1 and the final 10.000 cells were analysed with respect to (A) duration of G_1_ as function of the growth rate in G_1_, calculated as the difference between volume at START and the birth volume divided by the duration of G_1_; and (B) volume at START as a function of G_1_ duration.
(PDF)

S3 Fig Correlations of G_1_ duration, the growth rate in G_1_ and volume at START in Model-2. A fast growing (glucose) culture was simulated with Model-2 and the final 10.000 cells were analysed with respect to (A) duration of G_1_ as function of the growth rate in G_1_, calculated as the difference between volume at START and the birth volume divided by the duration of G_1_; and (B) volume at START as a function of G_1_ duration.
(PDF)

S4 Fig Parameter correlations. Parameter correlations derived from 100 fits started with uniformly distributed parameters within the parameter boundaries (axis ranges) for Model-1 (red) and Model-2 (blue).
(PDF)

S5 Fig Convergence of the objective value during parameter estimation. Shown is the evolution of the objective values (thin lines) and the mean objective value (thick lines) over the number of iterations for 100 rounds of parameter estimation for Model-1 (red) and Model-2 (blue), respectively. Note that in every iteration of the parameter estimation, the algorithm runs through a population of 12 different parameter sets for each model. The objective values displayed in the graph correspond to the best out of the 12 parameter sets simulated in every iteration (see parameter estimation algorithm for details).
(PDF)

S6 Fig Parameter distributions. Distribution of parameter and objective values derived from 100 fits started with uniformly distributed parameters within the parameter boundaries for Model-1 (red) and Model-2 (blue). X-axis ranges corresponds to parameter boundaries.
(PDF)

S7 Fig Correlations of the bud-volume at division and the growth rate in the budded phase (S-G_2_-M). Fast growing (glucose) cultures were simulated with Model-1 (red) and Model-2 (blue) and the final 10.000 cells were analysed, respectively. Shown is the bud-volume at division as a function of the growth rate in the budded phase (S-G_2_-M), calculated as the difference between volume at division and the volume at START divided by the duration of the budded phase (S-G_2_-M). Lines (Model-1: red; Model-2: blue) indicate least-squares regressions with respective correlation coefficient (R) and coefficient of determination (R^2^).
(PDF)

S8 Fig Correlations of the bud-volume at division and the budded phase duration (S-G_2_-M). Fast growing (glucose) cultures were simulated with Model-1 (red) and Model-2 (blue) and the final 10.000 cells were analysed, respectively. Shown is the bud-volume at division as a function of the duration of the budded phase (sum of S, G_2_ and M phase duration). Lines (Model-1: red; Model-2: blue) indicate least-squares regressions with respective correlation coefficient (R) and coefficient of determination (R^2^).
(PDF)

S9 Fig Correlations of the budded phase duration (S-G_2_-M) and the growth rate in the budded phase (S-G_2_-M). Fast growing (glucose) cultures were simulated with Model-1 (red) and Model-2 (blue) and the final 10.000 cells were analysed, respectively. Shown is the duration of the budded phase (sum of S, G_2_ and M phase duration) as a function of the growth rate in the budded phase (S-G_2_-M), calculated as the difference between volume at division and the volume at START divided by the duration of the budded phase (S-G_2_-M). Lines (Model-1: red; Model-2: blue) indicate least-squares regressions with respective correlation coefficient (R) and coefficient of determination (R^2^).
(PDF)

S10 Fig Generation time distributions for different genealogical ages for fast growing *in silico* cells. Shown are distributions of generation times (duration of one cell cycle) for fast growing cells (glucose) of the entire cell culture (all), daughters only (age 0), mothers of different ages (ages 1–6) and the sum of all mothers (mothers) for Model-1 (red medians) and Model-2 (blue medians). Values of the entire cell culture (all) for Model-1 (red) and Model-2 (blue) are also indicated (median, mean and mean calculated for the log-transformed distributions).
(PDF)

S11 Fig Generation time distributions for different genealogical ages for slow growing *in silico* cells. Shown are distributions of generation times (duration of one cell cycle) for slow growing cells (ethanol) of the entire culture (all), daughters only (age 0), mothers of different ages (ages 1–6) and the sum of all mothers (mothers) for Model-1 (red medians) and Model-2 (blue medians). Values of the entire cell culture (all) for Model-1 (red) and Model-2 (blue) are also indicated (median, mean and mean calculated for the log-transformed distributions).
(PDF)

S12 Fig Division ratios for different genealogical ages for slow growing *in silico* cells. Volume fraction of cell and bud at division is shown as a function of replicative age for data from experiments [41] (black dots) and from simulations of slow growing cells (ethanol) with Model-1 (red squares) and Model-2 (blue squares).
(PDF)

S13 Fig Generation time distributions for different genealogical ages for *in silico* cells with *CLN* overexpression. Shown are distributions of generation times (duration of one cell cycle) for fast growing cells (glucose) with *CLN* overexpression of the entire culture (all), daughters only (age 0), mothers of different ages (ages 1–6) and the sum of all mothers (mothers) for Model-1 (red medians) and Model-2 (blue medians). Values of the entire cell culture (all) for Model-1 (red) and Model-2 (blue) are also indicated (median, mean and mean calculated for the log-transformed distributions).
(PDF)

S14 Fig Cell cycle phase durations and cell size at birth, START and division for *in silico* cells with and without *CLN* overexpression. Shown are culture averages of G_1_ and S-G_2_-M durations, and the average volume at birth, at START and at budding for fast growing wild type (WT, solid empty bars) and *CLN* over producing (*oCLN*, dashed empty bars) cells. Averages for mother (green) and daughter (cyan) sub populations are indicated as well.
(PDF)

S1 Table Model statistics and ranking. The values of the best fit and the average over 100 fits are given for the objective value (wRSS), the log-likelihood (*ln(L(p))*) and the Akaike Information Criterion (AIC), as defined in Materials and Methods. The model rank is shown as well.
(PDF)

S1 Text The parameter estimation algorithm. A detailed description of the custom evolutionary parameter estimation algorithm is provided.
(PDF)

S1 File Single cell Model-2 in SBML format. Model-2 is provided in the systems biology markup language (SBML [63]) format to track the trajectories for a single newborn cell in any SBML standard compliant software. The model in the SBML file is set to show the time evolution of the mother line, i.e. properties of the bud are only followed as long as it is attached to the main cell.
(XML)

S2 File Model-1 and Model-2 within the cell populations simulator. A script is provided, which includes our core algorithm implemented in python to simulate Model-1 and Model-2 within an asynchronously growing population, i.e. following not only a single mother or daughter line, but all emerging cells simultaneously. The script can be used to simulate complete pedigrees of growing and dividing cells from single progenitor cells and contains routines to create basic plots for analysis of the resulting cell population. The model parameters, plots, final numbers of cells within the population and other properties can be edited directly in the script.
(ZIP)
