# Supplementary material for: Bud-Localization of CLB2 mRNA Can Constitute a Growth Rate Dependent Daughter Sizer
Source: PLoS Comput Biol. 2015 Apr 24;11(4):e1004223. doi: 10.1371/journal.pcbi.1004223 (PMC4429581; doi:10.1371/journal.pcbi.1004223)
Supplement: S1 Fig — A population of fast growing cells (glucose, final number: 10.000) was simulated with Model-2 for analysis. (A) Population size homeostasis emerges through the dynamics of all individual cells (thin coloured lines). After division cells restart at lower volumes. Since always two cells result from one dividing cell, thin lines become denser over time. The thick dark blue line indicates the average cell size in the population and the light-blue area the range of one standard deviation (SD). Despite the diverse behaviour of the individual trajectories, average and SD stabilise quickly when the population grows starting from one individual at time 0 minutes. (B) Volume at START correlates with the growth rate in G1 for individual cells in the model. Growth rate in G1 was calculated as the difference between volume at START and the birth volume divided by the duration of G1. Black line indicates a least-squares regression with correlation coefficient (R) and coefficient of determination (R2). (PDF) [file pcbi.1004223.s001.pdf]

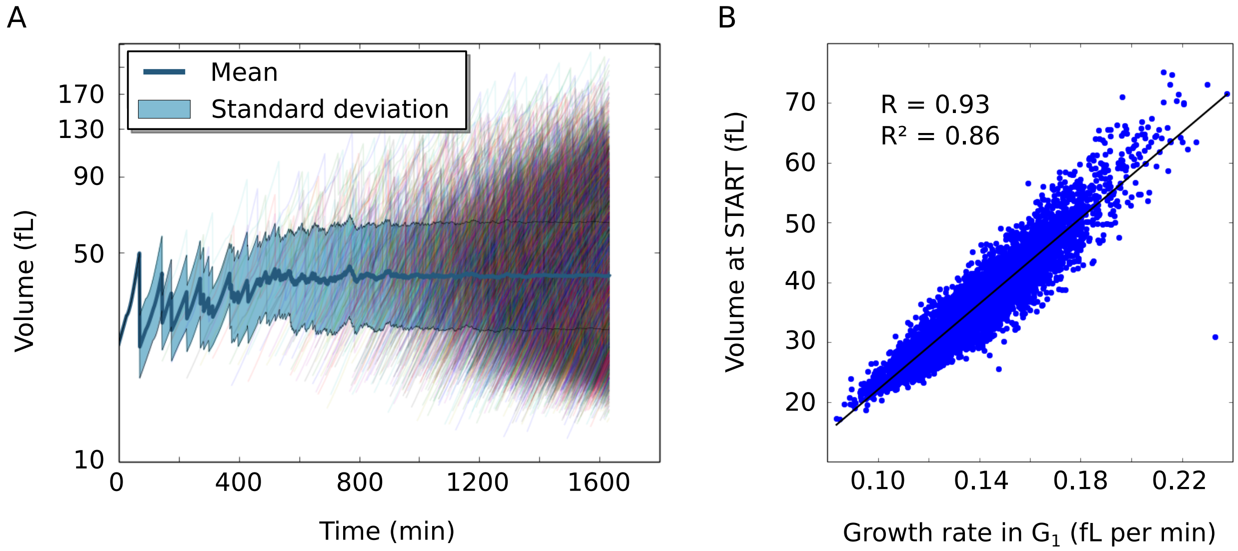

Figure S1: Population size homeostasis through growth rate dependent size control in G_1 (Model-2). A population of fast growing cells (glucose, final number: 10.000) was simulated with Model-2 for analysis. (A) Population size homeostasis emerges through the dynamics of all individual cells (thin colored lines). After division cells restart at lower volumes. Since always two cells result from one dividing cell, thin lines become denser over time. The thick dark blue line indicates the average cell size in the population and the light-blue area the range of one standard deviation (SD). Despite the diverse behavior of the individual trajectories, average and SD stabilize quickly when the population grows starting from one individual at time 0 minutes. (B) Volume at START correlates with the growth rate in G_1 for individual cells in the model. Growth rate in G_1 was calculated as the difference between volume at START and the birth volume divided by the duration of G_1. Black line indicates a least-squares regression with correlation coefficient (R) and coefficient of determination (R^2).
