# Supplementary material for: Bud-Localization of CLB2 mRNA Can Constitute a Growth Rate Dependent Daughter Sizer
Source: PLoS Comput Biol. 2015 Apr 24;11(4):e1004223. doi: 10.1371/journal.pcbi.1004223 (PMC4429581; doi:10.1371/journal.pcbi.1004223)
Supplement: S2 Fig — A fast growing (glucose) culture was simulated with Model-1 and the final 10.000 cells were analysed with respect to (A) duration of G1 as function of the growth rate in G1, calculated as the difference between volume at START and the birth volume divided by the duration of G1; and (B) volume at START as a function of G1 duration. (PDF) [file pcbi.1004223.s002.pdf]

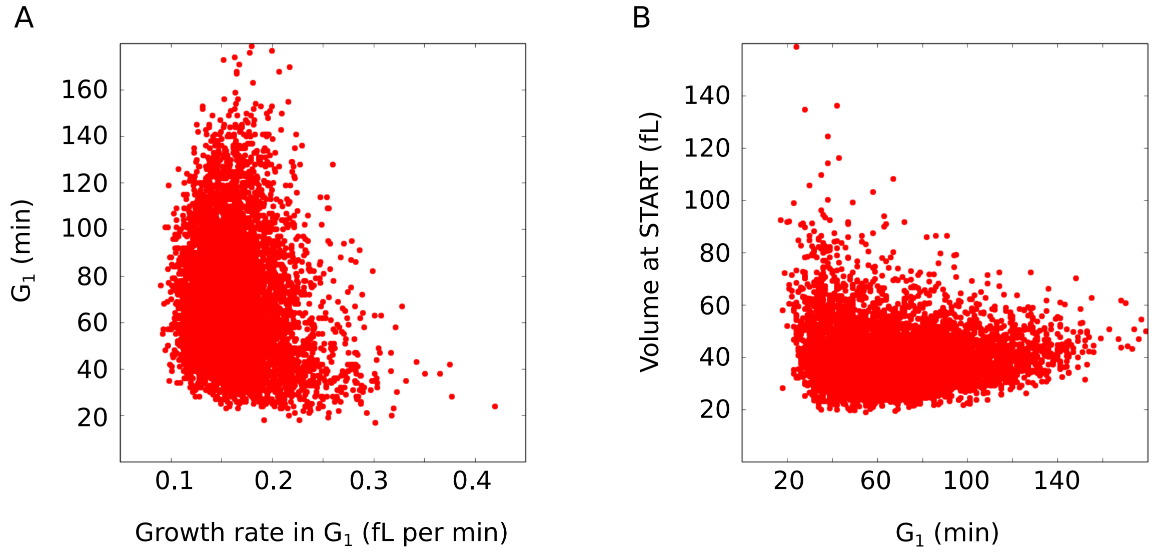

Figure S2: Correlations of G_1 duration, the growth rate in G_1 and volume at START in Model-1. A fast growing (glucose) culture was simulated with Model-1 and the final 10.000 cells were analysed with respect to (A) duration of G_1 as function of the growth rate in G_1, calculated as the difference between volume at START and the birth volume divided by the duration of G_1; and (B) volume at START as a function of G_1 duration.
