# Supplementary material for: Bud-Localization of CLB2 mRNA Can Constitute a Growth Rate Dependent Daughter Sizer
Source: PLoS Comput Biol. 2015 Apr 24;11(4):e1004223. doi: 10.1371/journal.pcbi.1004223 (PMC4429581; doi:10.1371/journal.pcbi.1004223)
Supplement: S4 Fig — Parameter correlations derived from 100 fits started with uniformly distributed parameters within the parameter boundaries (axis ranges) for Model-1 (red) and Model-2 (blue). (PDF) [file pcbi.1004223.s004.pdf]

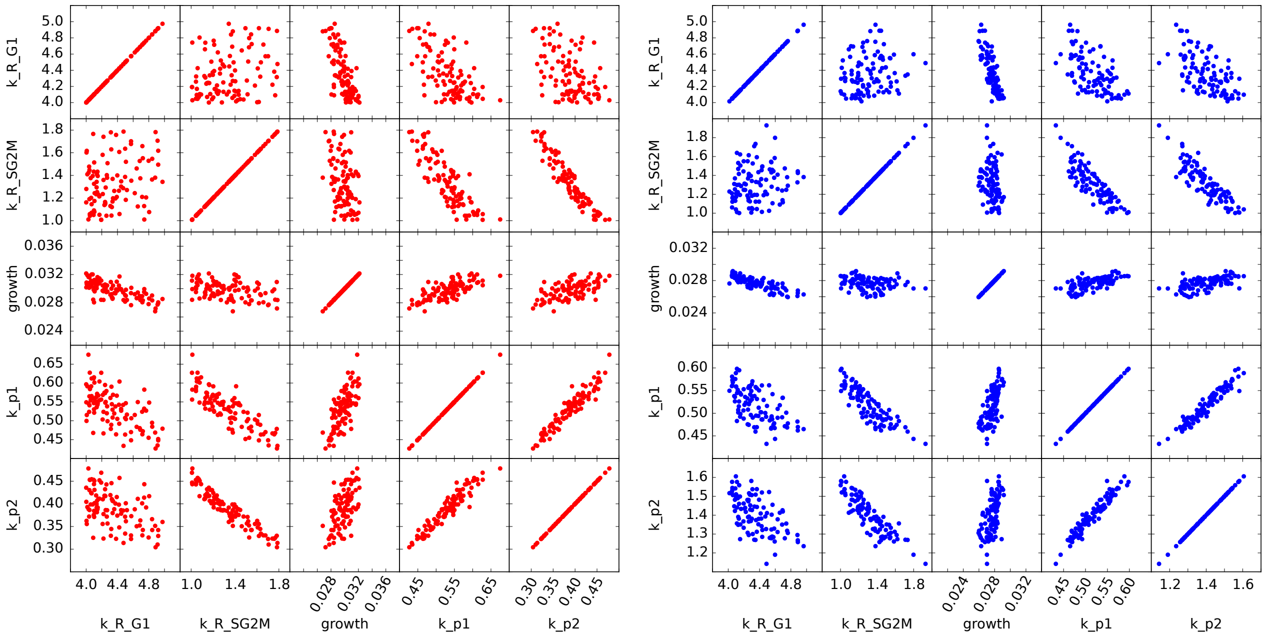

Figure S4: Parameter correlations. Parameter correlations derived from 100 fits started with uniformly distributed parameters within the parameter boundaries (axis ranges) for Model-1 (red) and Model-2 (blue).
