# Supplementary material for: Bud-Localization of CLB2 mRNA Can Constitute a Growth Rate Dependent Daughter Sizer
Source: PLoS Comput Biol. 2015 Apr 24;11(4):e1004223. doi: 10.1371/journal.pcbi.1004223 (PMC4429581; doi:10.1371/journal.pcbi.1004223)
Supplement: S5 Fig — Shown is the evolution of the objective values (thin lines) and the mean objective value (thick lines) over the number of iterations for 100 rounds of parameter estimation for Model-1 (red) and Model-2 (blue), respectively. Note that in every iteration of the parameter estimation, the algorithm runs through a population of 12 different parameter sets for each model. The objective values displayed in the graph correspond to the best out of the 12 parameter sets simulated in every iteration (see parameter estimation algorithm for details). (PDF) [file pcbi.1004223.s005.pdf]

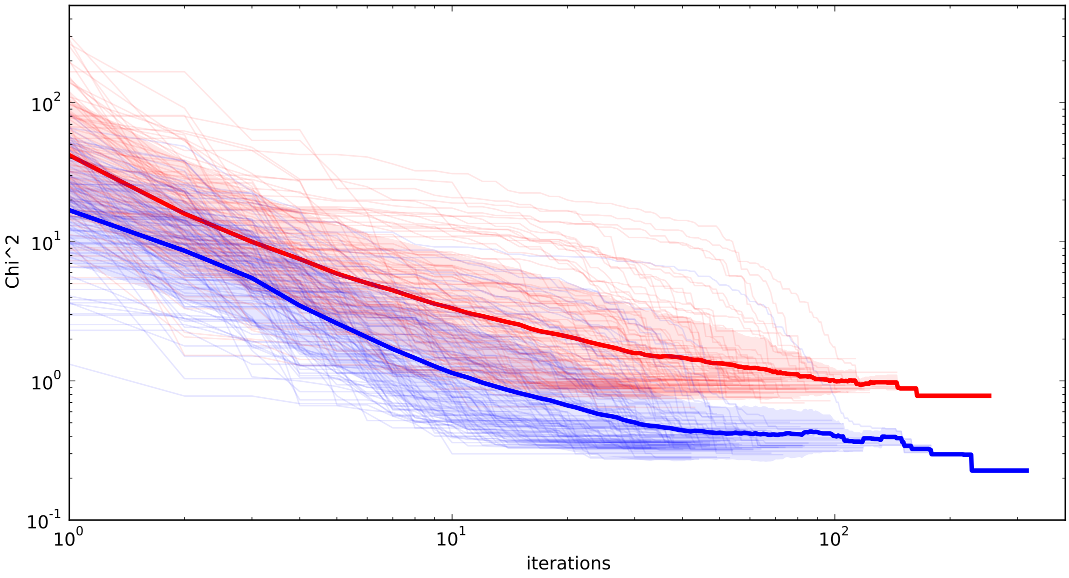

Figure S5: Convergence of the objective value during parameter estimation. Shown is the evolution of the objective values (thin lines) and the mean objective value (thick lines) over the number of iterations for 100 rounds of parameter estimation for Model-1 (red) and Model-2 (blue), respectively. Note that in every iteration of the parameter estimation, the algorithm runs through a population of 12 different parameter sets for each model. The objective values displayed in the graph correspond to the best out of the 12 parameter sets simulated in every iteration (see parameter estimation algorithm for details).
