# Supplementary material for: Bud-Localization of CLB2 mRNA Can Constitute a Growth Rate Dependent Daughter Sizer
Source: PLoS Comput Biol. 2015 Apr 24;11(4):e1004223. doi: 10.1371/journal.pcbi.1004223 (PMC4429581; doi:10.1371/journal.pcbi.1004223)
Supplement: S9 Fig — Fast growing (glucose) cultures were simulated with Model-1 (red) and Model-2 (blue) and the final 10.000 cells were analysed, respectively. Shown is the duration of the budded phase (sum of S, G2 and M phase duration) as a function of the growth rate in the budded phase (S-G2-M), calculated as the difference between volume at division and the volume at START divided by the duration of the budded phase (S-G2-M). Lines (Model-1: red; Model-2: blue) indicate least-squares regressions with respective correlation coefficient (R) and coefficient of determination (R2). (PDF) [file pcbi.1004223.s009.pdf]

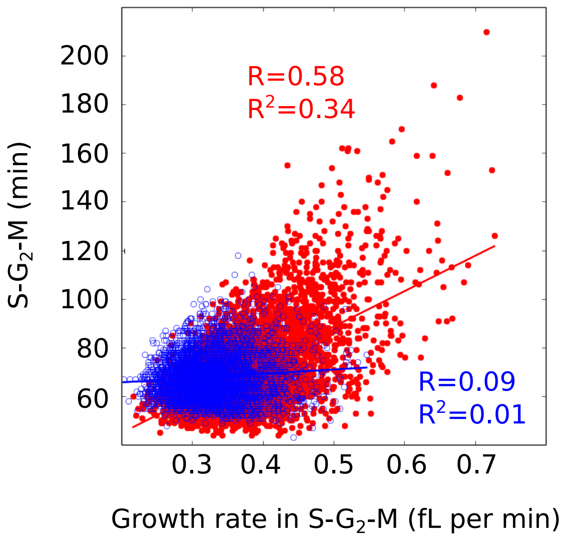

Figure S9: Correlations of the budded phase duration (S-G_2-M) and the growth rate in the budded phase (S-G_2-M). Fast growing (glucose) cultures were simulated with Model-1 (red) and Model-2 (blue) and the final 10.000 cells were analysed, respectively. Shown is the duration of the budded phase (sum of S, G_2 and M phase duration) as a function of the growth rate in the budded phase (S-G_2-M), calculated as the difference between volume at division and the volume at START divided by the duration of the budded phase (S-G_2-M). Lines (Model-1: red; Model-2: blue) indicate least-squares regressions with respective correlation coefficient (R) and coefficient of determination (R^2).
