# Supplementary material for: Bud-Localization of CLB2 mRNA Can Constitute a Growth Rate Dependent Daughter Sizer
Source: PLoS Comput Biol. 2015 Apr 24;11(4):e1004223. doi: 10.1371/journal.pcbi.1004223 (PMC4429581; doi:10.1371/journal.pcbi.1004223)
Supplement: S10 Fig — Shown are distributions of generation times (duration of one cell cycle) for fast growing cells (glucose) of the entire cell culture (all), daughters only (age 0), mothers of different ages (ages 1–6) and the sum of all mothers (mothers) for Model-1 (red medians) and Model-2 (blue medians). Values of the entire cell culture (all) for Model-1 (red) and Model-2 (blue) are also indicated (median, mean and mean calculated for the log-transformed distributions). (PDF) [file pcbi.1004223.s010.pdf]

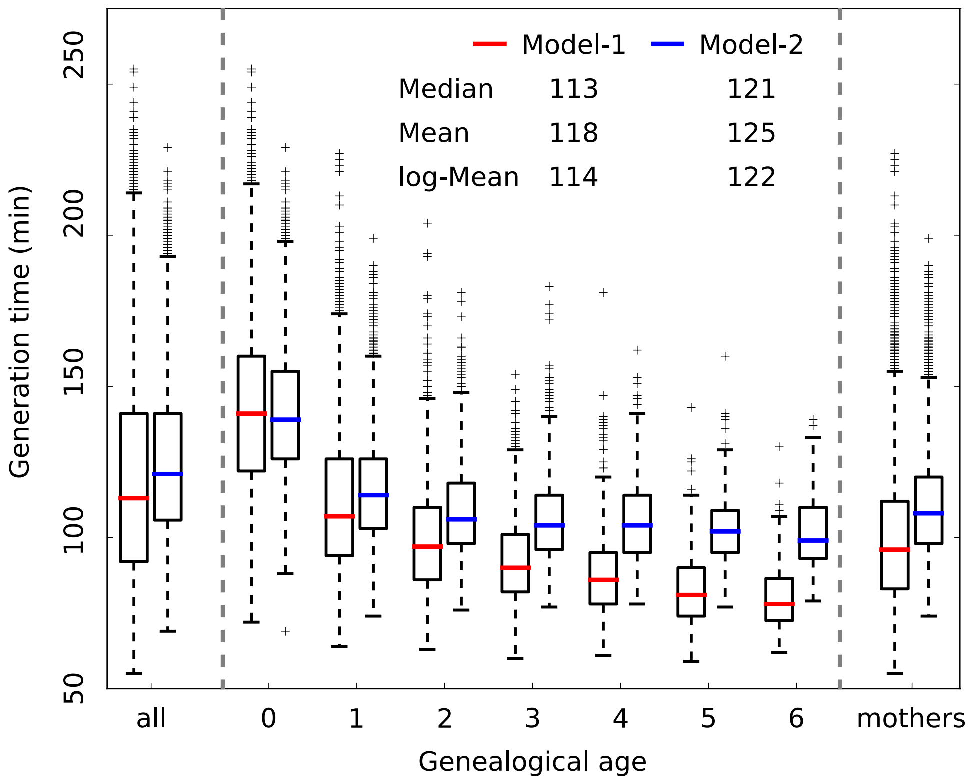

Figure S10: Generation time distributions for different genealogical ages for fast growing *in silico* cells. Shown are distributions of generation times (duration of one cell cycle) for fast growing cells (glucose) of the entire cell culture (all), daughters only (age 0), mothers of different ages (ages 1-6) and the sum of all mothers (mothers) for Model-1 (red medians) and Model-2 (blue medians). Values of the entire cell culture (all) for Model-1 (red) and Model-2 (blue) are also indicated (median, mean and mean calculated for the log-transformed distributions).
