# Supplementary material for: Bud-Localization of CLB2 mRNA Can Constitute a Growth Rate Dependent Daughter Sizer
Source: PLoS Comput Biol. 2015 Apr 24;11(4):e1004223. doi: 10.1371/journal.pcbi.1004223 (PMC4429581; doi:10.1371/journal.pcbi.1004223)
Supplement: S12 Fig — Volume fraction of cell and bud at division is shown as a function of replicative age for data from experiments [41] (black dots) and from simulations of slow growing cells (ethanol) with Model-1 (red squares) and Model-2 (blue squares). (PDF) [file pcbi.1004223.s012.pdf]

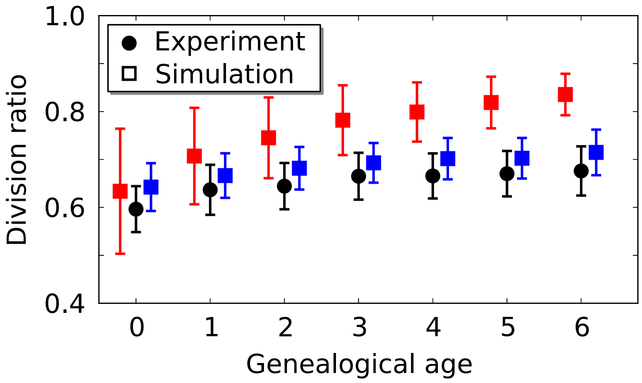

Figure S12: Division ratios for different genealogical ages for slow growing *in silico* cells. Volume fraction of cell and bud at division is shown as a function of replicative age for data from experiments [1] (black dots) and from simulations of slow growing cells (ethanol) with Model-1 (red squares) and Model-2 (blue squares).

[1] Natalie A Cookson, Scott W Cookson, Lev S Tsimring, and Jeff Hasty. Cell cycle-dependent variations in protein concentration. *Nucleic Acids Research*, 38(8):2676–2681, December 2009.
