# Supplementary material for: Bud-Localization of CLB2 mRNA Can Constitute a Growth Rate Dependent Daughter Sizer
Source: PLoS Comput Biol. 2015 Apr 24;11(4):e1004223. doi: 10.1371/journal.pcbi.1004223 (PMC4429581; doi:10.1371/journal.pcbi.1004223)
Supplement: S14 Fig — Shown are culture averages of G1 and S-G2-M durations, and the average volume at birth, at START and at budding for fast growing wild type (WT, solid empty bars) and CLN over producing (oCLN, dashed empty bars) cells. Averages for mother (green) and daughter (cyan) sub populations are indicated as well. (PDF) [file pcbi.1004223.s014.pdf]

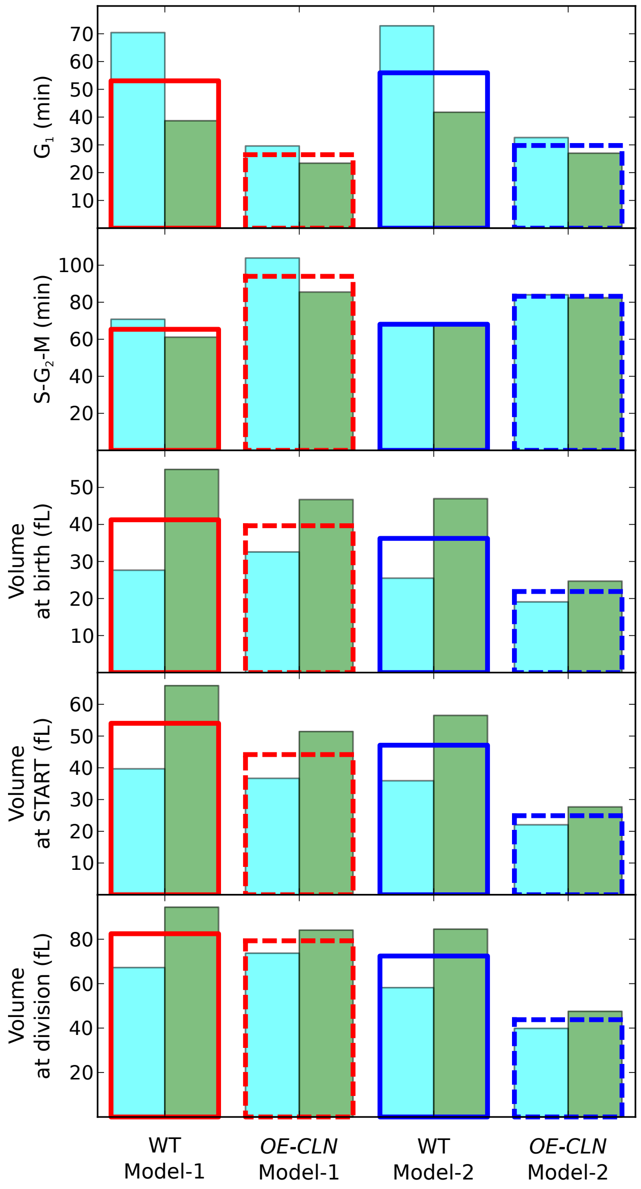

Figure S14: Cell cycle phase durations and cell size at birth, START and division for *in silico* cells with and without *CLN* overexpression. Shown are culture averages of G_1 and S-G_2-M durations, and the average volume at birth, at START and at budding for fast growing wild type (WT, solid empty bars) and *CLN* over producing (*oCLN*, dashed empty bars) cells. Averages for mother (green) and daughter (cyan) subpopulations are indicated as well.
