# Supplementary material for: Bud-Localization of CLB2 mRNA Can Constitute a Growth Rate Dependent Daughter Sizer
Source: PLoS Comput Biol. 2015 Apr 24;11(4):e1004223. doi: 10.1371/journal.pcbi.1004223 (PMC4429581; doi:10.1371/journal.pcbi.1004223)
Supplement: S1 Table — The values of the best fit and the average over 100 fits are given for the objective value (wRSS), the log-likelihood (ln(L(p))) and the Akaike Information Criterion (AIC), as defined in Materials and Methods. The model rank is shown as well. (PDF) [file pcbi.1004223.s015.pdf]

Table S1: Model statistics and ranking

	Model-1	Model-2
wRSS (best)	0.65	0.23
wRSS (average)	0.96	0.37
ln(L(p)) (best)	-25.87	-25.66
ln(L(p)) (average)	-26.02	-25.73
AIC (best)	61.74	61.32
AIC (average)	62.05	61.47
Rank	2	1

The values of the best fit and the average over 100 fits are given for the objective value (wRSS), the log-likelihood (*ln(L(p))*) and the Akaike Information Criterion (AIC). The model rank is shown as well.
