# Supplementary material for: Bud-Localization of CLB2 mRNA Can Constitute a Growth Rate Dependent Daughter Sizer
Source: PLoS Comput Biol. 2015 Apr 24;11(4):e1004223. doi: 10.1371/journal.pcbi.1004223 (PMC4429581; doi:10.1371/journal.pcbi.1004223)
Supplement: S1 Text — A detailed description of the custom evolutionary parameter estimation algorithm is provided. (PDF) [file pcbi.1004223.s016.pdf]

Supplementary Text on Parameter Estimation

to

Bud-localization of *CLB2* mRNA can constitute a growth rate dependent daughter sizer

by

Thomas W. Spiesser, Clemens Kühn, Marcus Krantz and Edda Klipp

SI. The Parameter Estimation Algorithm

The genetic algorithm employed to estimate parameters for Model-1 and Model-2 is implemented in python and available upon request. In the current implementation the model simulation and the computation of objective value are hard-coded into the algorithm, but the implementation can be easily adapted by exchanging the respective functions (e.g. to run parameter estimation for single cell models, different cell types or completely different kinds of models like BioNetGen models).

For convenience, we use a class `Paramest` that holds all information needed for the algorithm. The structure of the algorithm is roughly:

```
initialize parameter sets and simulate;
while not reached abort criteria do
    construct new parameter sets by mating;
    simulate model with new parameter sets;
    compute objective value for each;
    remove lowest ranking parameter sets;
    output best with objective value;
    update convergence criteria;
    continue until convergence or abort criteria reached;
end
```

Algorithm 1: The runfit main loop.

In the following, we will explain each line of Algorithm 1 and the `Paramest` class.

SI.1. Requirements

Before running the algorithm, the following are required:

- a dictionary of boundaries for each parameter
- the data for computation of the objective value
- a function for **simulation** of the model
- a function for computation of the **objective value**

To apply this algorithm to different estimation tasks, the functions for **simulation** and computation of the **objective value** must be adapted to the specific task.

SI.2. The `Paramest` class

The `Paramest` class holds the following:

- `boundaries` for the parameters
- the `data` for computation of the objective value
- the number of `CPUs` to use for parallel computation (default: 1)
- `convergence_threshold`: max. number of iterations without improve of fit (default: 5)
- the number of models with different parameter sets (`popsize`; default: 10)

- the maximum number of iterations (`maxiter`; default: 1000000)
- a continuously updated list of the current parameter sets including the respective objective value: `currentpop`
- evolution of the fit; best parameter set and objective value for every iteration (`fit`)
- `iterations` counter
- a list indicating the strength of parameter perturbations `randlist` (see **Mating**)
- the index to `randlist`, `randlistcounter`
- an indicator of how many simulations to perform for an objective value in case of stochastic models, `runsformean`.

The datatype of `data` is not specified, but it should suit the type expected for the computation of the objective value (see function: `computeOV`).

SI.3. Initialize

```
foreach Parameter in boundaries do
    if upperBound{parameter}!=lowerBound{parameter} then
        sample new value from uniform distribution between upper and lower boundary
    end
end
```

Algorithm 2: The initialization loop. Sampling is performed in log-space whenever parameter boundaries do not include zero.

SI.4. Simulate

The simulate function calls the model to be simulated. Here, the ODE solver or any other method is to be executed. For Model-1 and Model-2, a function for simulation of the cell populations is called with

- final number of cells
- initial cell number
- specific parameter set.

In the case of stochastic models, simulations can be repeated (`runsformean`).

```
foreach parameter set in currentpop do
    for i in range(1, runsformean) do
        simulate(Model[parameter set])
    end
end
```

Algorithm 3: The simulation loop.

SI.5. Objective Value

Custom function to compute the objective value (`ov`) for each parameter set. In the present case, the weighted sum of squared residuals was computed between experimental [1] and simulated data concerning

- G1 duration of daughter cells grown in glucose
- budded phase duration of daughter cells grown in glucose
- volume at birth of daughter cells grown in glucose
- volume at START of daughter cells grown in glucose
- G1 duration of daughter cells grown in ethanol
- budded phase duration of daughter cells grown in ethanol
- volume at birth of daughter cells grown in ethanol
- volume at START of daughter cells grown in ethanol.

SI.6. Remove

```
MedianOV ← Median(currentpop{ov});
newpop ← [];
insert into newpop all entries from currentpop where ov ≤ MedianOV
```

Algorithm 4: The removal of lowest ranking parameter sets.

SI.7. Output

The algorithms writes the best obejctive value, the according parameters, the mean objective value and the standard deviation of obejctive values to `stdout` during each iteration.

SI.8. Mating

```
OVs← currentpop[i]{ov};
// assign a probability to every parameter set depending on the ov
rProbs←sum(OVs)/OVs[i];
tProbs← rProbs[i]/sum(rProbs);
foreach parmeter set in currentpop do
    assign partner based on tProbs;
    newparset ← parmeter set;
    foreach parameter in newparset do
        // set each parameter to the value of either the partner or the
           mean
        dice ← randint(3);                    // randint(3) is either 0,1,2
        if dice==0 then parameter←parameter;
        else if dice==1 then
        | parameter←currentpop[partner]{parameter}
        end
        else if dice==2 then
        | parameter←mean(parameter,currentpop[partner]{parameter})
        end
        // wiggle → perturb parameter value with random number from normal
           distribution (μ=0) and increasing σ according to randlist
        parameter←parameter+random()*randlist[randlistcounter]*parameter;
        check if parameter is still within boundaries;
    end
end
```

Algorithm 5: Mating of parameter sets.

New parameter sets are created through a combination of mating and perturbations. Probabilities for mating (tProbs) are assigned to every parameter set. The probability for mating is high when the objective value is low. Whether the new parameter set inherits the values from the current set, its mating partner or the mean of both occurs by chance (dice). Lastly, the inherited parameter values are perturbed proportional to their value and the state of the fit (randlist, see textbfDetailed main loop).

SI.9. Detailed main loop

In mating, the 'wiggling' step introduces stochasticity into the procedure. The magnitude of the stochasiticity is controlled by the current position randlistcounter in randlist. The values in randlist go from small to large and randlistcounter is reset whenever a new best obejctive value is found to scan the immediate neighbourhood. If no improvement in objective value is found in convergencecounter iterations, randlistcounter is increased by 1. The algorithm aborts when either *maxiter* iterations were run or randlistcounter exceeds the size of randlist. To illustrate this, a more detailed representation of the main loop is shown in Algorithm 6.

SI.10. Parameters of the algorithm

Whether and how fast this algorithm converges to a unique solution crucially depends on the problem and the contents of randlist and convergencecounter. As these eventually also control the number of iterations the algorithm computes, it is sensible to check the convergence

```
while iterations≤maxiter do
    iterations++;
    if randlistcounter≤len(randlist) then
        mate();
        simulate();
        objective_value();
        remove();
        newbestov← min(currentpop[ov]);
        if oldbestOV/newbestOV > 1 then
            randlistcounter←0;
        end
        if fit[-convergencecounter]{bestov}= newbestov then
            randlistcounter++;
        end
        output
    end
    else
        break
    end
end
```

Algorithm 6: Main outer loop and control of aborting conditions

behavior of the algorithm by comparing the final results of multiple fits and the progress within each fit.

References

[1] Francisco Ferrezuelo, Neus Colomina, Alida Palmisano, Eloi Garí, Carme Gallego, Attila Csikász-Nagy, and Martí Aldea. The critical size is set at a single-cell level by growth rate to attain homeostasis and adaptation. *Nature Communications*, 3:1012, January 2012.
